# Severe dengue–related deaths in the elderly population soared in Southern Brazil in 2024

**DOI:** 10.1016/j.ijregi.2025.100577

**Published:** 2025-01-25

**Authors:** Alexandre Sarmento Queiroga, Danielly Alves Mendes Barbosa, Tulio de Lima Campos, Alexandre Vargas Schwarzbold, Andre M. Siqueira, Richard Steiner Salvato, Gabriel Luz Wallau

**Affiliations:** 1 Núcleo de Bioinformática, Instituto Aggeu Magalhães (IAM), Fundação Oswaldo Cruz (Fiocruz), Recife, Brazil; 2 Departamento de Entomologia, Instituto Aggeu Magalhães (IAM), Fundação Oswaldo Cruz (Fiocruz), Recife, Brazil; 3 Centro de Ciências da Saúde, Universidade Federal de Santa Maria (UFSM), Santa Maria, Brazil; 4 Drugs for Neglected Diseases Initiative (DNDi), Rio de Janeiro, RJ, Brazil; and Instituto Nacional de Infectologia Evandro Chagas, Fundação Oswaldo Cruz, Rio de Janeiro, Brazil; 5 Programa de Pós-Graduação em Biociências, Universidade Federal de Ciências da Saúde de Porto Alegre, Porto Alegre, Brazil; 6 Universidade Federal de Santa Maria (UFSM), Santa Maria, Brazil; 7 Department of Arbovirology and Entomology, Bernhard Nocht Institute for Tropical Medicine, WHO Collaborating Center for Arbovirus and Hemorrhagic Fever Reference and Research. National Reference Center for Tropical Infectious Diseases, Bernhard-Nocht-Straße, Germany

**Keywords:** Arbovirus, Increasing burden, Vulnerability

## Abstract

**Introduction:**

Dengue fever is expanding worldwide on the track of climate change. In 2024, more than 14 million cases were reported. Around 6.5 million of those were reported in Brazil alone, reaching a staggering number of around 6000 deaths. Dengue lethality in severe cases were particularly high in Rio Grande do Sul, a historically less affected state which has witnessed an increased incidence and outbreaks in the last 4 years.

**Design:**

In this report, we raised some hypotheses to explain the high lethality.

**Results:**

Rio Grande do Sul has the highest proportion of elderly individuals among all states in Brazil. This factor, associated with the immunologically naive population, low levels of awareness to detect and treat severe dengue, and the difficulties in managing elderly patients, may have contributed to this higher lethality in severe dengue.

**Conclusions:**

The expected increasing dengue incidence in the region in the subsequent years highlights the urgent need of an integrated approach to raise awareness, reduce mosquito populations, and reduce dengue burden in the region, particularly, in the most vulnerable elderly population.

## Highlights

• There is an ongoing increase in dengue in a subtropical region that was historically much less affected.
• In 2024, the number of severe cases and deaths increased substantially.
• The lethality rate in the elderly population with severe dengue is higher.
• Demographic differences and lack of awareness may explain the higher lethality.
• Preparedness is key to reduce the dengue burden in elderly vulnerable groups next season.

**Figure 1:**
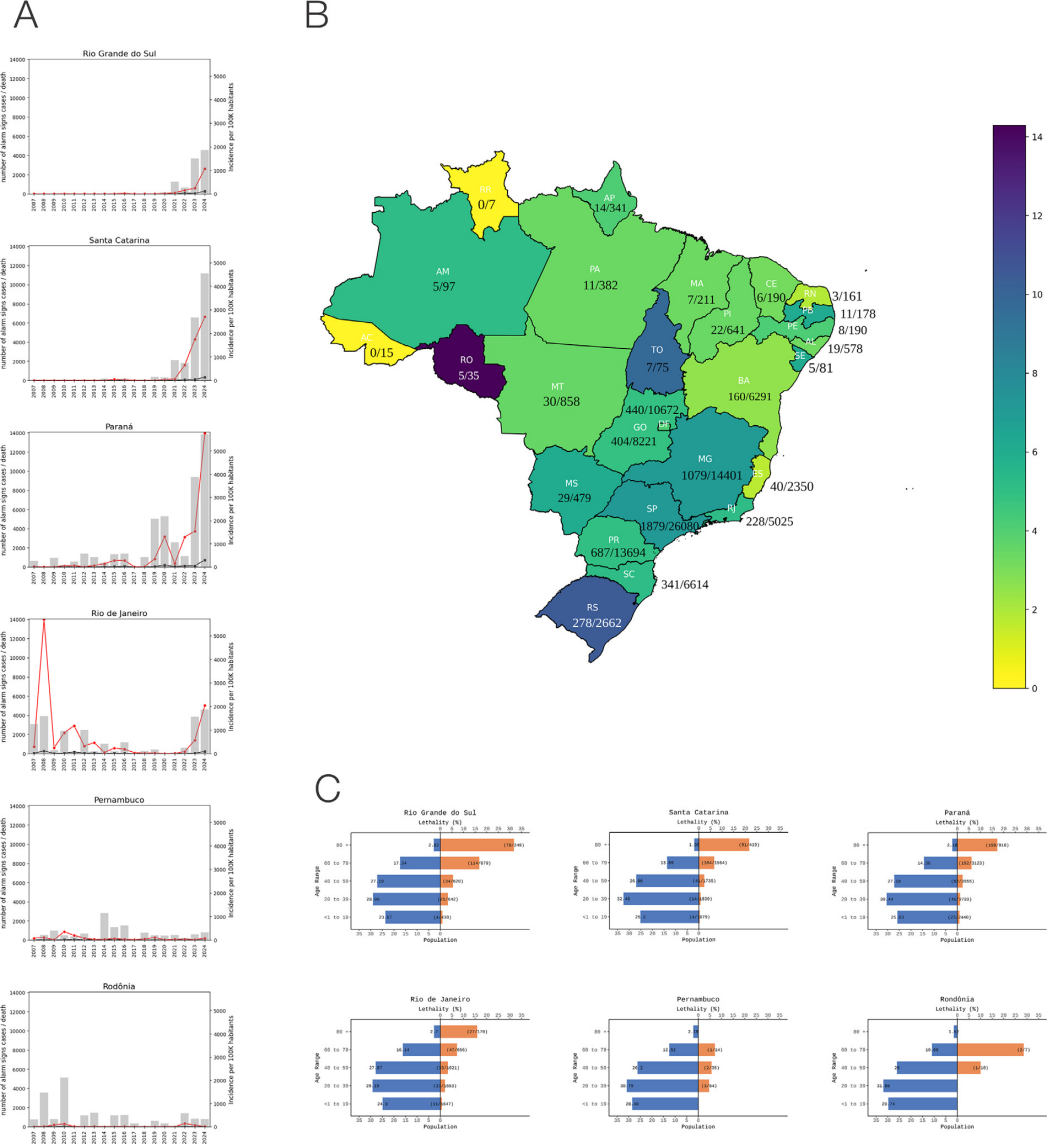
Dengue incidence and lethality in different regions of Brazil and epidemiologic profile of cases exhibiting warning signs of severe dengue. (a) From top to bottom, the incidence per 100,000 habitants, severe dengue, and lethality in severe dengue cases in Rio Grande do Sul (RS), Santa Catarina (SC), Paraná (PR), Rio de Janeiro (RJ), Pernambuco (PE), and Rondônia (RO). The bars represent the incidence and the line represents the total numbers of severe dengue (red) and deaths in severe dengue cases (black) per year. (b) The heat map of lethality in severe dengue cases per Brazilian state in 2024. RS is the southernmost state of Brazil. In addition to each name of the state's abbreviation, there is the number of deaths in severe dengue cases/number of cases with warning signs. (c) The pyramid age of RS, Santa Catarina (SC), PR, RJ, PE, and RO. The blue bars represent the proportion of the population per age group, whereas the orange horizontal bars represent the proportion of deaths in severe dengue cases (lethality) per age in each state; over the orange bars, there is also the number of deaths in severe dengue cases/number of severe dengue cases.

Dengue virus is endemic in tropical regions of the world, causing around 100-400 million cases and 4000-5000 deaths per year [1]. This virus, the *Orthoflavivirus denguei*, is mainly transmitted in the urban cycle by the anthropophilic mosquitoes *Aedes aegypti* and *Aedes albopictus* [2,3]. In Brazil, dengue virus has been monitored using molecular tools since 1986, and the four serotypes have been detected and are known to circulate in the country [4]. The annual dengue incidence has increased country-wide since then, except for the southern three subtropical Brazilian states where transmission has been limited due to climatic barriers that slowed down the spread of the vector in this region [5]. However, on the track of climate change, the *Aedes aegypti* mosquito slowly spread to southern areas due to the erosion of the climatic barrier, leading to new outbreaks in immunologically naive human populations. Data-driven models suggest that dengue transmission risk by *A. aegypti* increases when temperature ranges from 21.3°C to 34.0°C, whereas *Aedes albopictus* transmission risk increases with temperature, varying between 19.9°C and 29.4°C [6,7]. The average annual temperature of Rio Grande do Sul (RS) currently ranges from 15°C to18.6°C; however, climatic models predictions from the Intergovernmental Panel of Climate Change points to an increase of 0.95°C per year in the next 70 years in annual average temperature in RS [8]. The expansion of dengue in this southern frontier suggests that an increased climate suitability and/or vector adaptation to thrive in regions with lower temperature are occurring and is associated with a higher risk of vector-borne disease circulation between Argentina, Paraguay, Uruguay, and Brazil [9]. This is the case in RS, a state historically much less affected, where dengue incidence was mostly associated with travelers from endemic tropical Brazilian states [10,11]. The first dengue autochthonous transmission in RS was detected in 2007; however, consistent detection of cases within the state only began in 2010 [11]. In the last 10 years (2015-2024), dengue incidence increased 9.45-fold in RS, compared with basal levels in the previous decade (2004-2014) (Figure 1a and Supplementary Table 1). In 2024, Brazil reached a historical record of notifications, around 6.5 million suspected dengue cases and 6000 deaths, and RS surpassed 202,658 suspected cases with at least 278 deaths until October 2024 (Figure 1a). More concerning, RS exhibited the second-highest lethality rate in severe dengue cases in Brazil (10.44), only surpassed by Rondônia, a state located in the Amazon region (14.29) (Figure 1a, Figure 1b). It is important to note that in 2024, the total number of deaths in severe dengue cases in Rondônia was three, whereas in RS, it reached 256. The state of Santa Catarina reached 254 deaths, whereas Paraná reached 500 (Figure 1a, Figure 1b). Rio de Janeiro reached 151 deaths in more than 5000 severe dengue cases and Pernambuco reached six deaths in 193 severe dengue cases (Figure 1a, Figure 1b). We shall highlight that Rio de Janeiro and Pernambuco are considered endemic for dengue for several decades, and, in 2024, it was the first time that deaths in severe dengue cases in Rio Grande Sul surpassed the numbers in these states. These staggering numbers led us to further explore the possible causes of such high lethality in RS. Deceased patients with severe dengue concentrated at ages above 60 years (193 of 928 patients with severe dengue in these age strata) in the state (Figure 1c, Supplementary Figure 1). We found no major differences in age strata of general population and lethality in dengue cases showing warning signs in selected RS cities (Supplementary Figure 2 and 3). The RS population has the highest average age among all Brazilian states [12]. Hence, the difficulty of treating the disease in a population with an increased number of comorbidities with lower resilience to dehydration and hyperhydration may contribute to more severe cases and increased chance of death affecting the elder age groups [13]. In addition, because this region had not been historically affected by dengue, there may be a lack of awareness among health professionals and the general population to timely suspect and manage dengue, as well as a limited preparation of the health systems to reduce the risk of disease progression. Mortality in severe dengue manifestation ranges from 0% to 2% with early treatment but over 10% if treatment is delayed [14]. Of note, high resolution genomic surveillance implemented in the state (GISAID EpiArbo) [15] allowed us to detect an ongoing serotype shift from dengue virus DENV1 to DENV2 in the state (2023: DENV1 = 100 genomes and DENV2 = 19 genomes) to 2024 (DENV1 = 176 genomes and DENV2 = 105 genomes), mirroring the trends observed in other Brazilian states, but no new lineage has emerged in the state recently. An increase in DENV2 serotype have been shown to yield contrasting results in terms of more severe dengue fever [[16], [17], [18]], as well as serotype shifts and secondary infection [19]; therefore, the increase in DENV2 and its ongoing serotype prevalence shift in the state may have contributed to the increase in severe dengue and deaths. However, during 2024, several other states experienced DENV2 infections and DENV1 to DENV2 transition (Pernambuco 2023: DENV1 = 24, DENV2 = 0 / 2024: DENV1 = 110, DENV2 = 90; 2023 Rio de Janeiro DENV1 = 40, DENV2 = 8 / 2024: DENV1 = 75, DENV2 = 58; Santa Catarina 2023: DENV1 = 152, DENV2 = 46 / 2024: DENV1 = 292, DENV2 = 249; Paraná 2023: DENV1 = 104, DENV2 = 5 / 2024: DENV1 = 100, DENV2 = 61) without a comparable increase in severe dengue deaths as the RS state, suggesting that the genetic background of the virus is not the most important factor to explain an increased lethality of dengue with warning signs in RS. In 2024, we witnessed a dangerous combination of increased incidence of dengue in an immunologically naive population with higher proportion of elderly individuals, likely associated with limited awareness of health professionals and the whole population regarding the importance of closely monitoring dengue warning signs to reduce dengue severe cases and deaths and, more specifically, highlighting the challenges of managing dengue in a more vulnerable population that is less resilient to fluid management strategies. Apart from highlighting a high risk of primary infections evolving severely in older individuals (differently from the usual expectation of higher risk of severity in secondary infections), our data raises awareness of what can happen when dengue transmission emerges in areas with a high proportion of elderly individuals without previous exposure. The difficulties in managing these individuals (currently not targeted by dengue vaccination strategies) also highlights the need for developing and delivering effective treatments against dengue that can reduce progression to severe disease, which would lead to individual and public health benefits for epidemic-prone areas. These results highlight the urgent need to protect the whole population, particularly, the most vulnerable elderly population, from severe dengue, especially given the expected increase of dengue burden in the southern region of Brazil.

## Declarations of competing interest

The authors have no competing interests to declare.

## Appendix Supplementary materials

Image, application 1

Image, application 2
