# Supplementary material for: Severe dengue–related deaths in the elderly population soared in Southern Brazil in 2024
Source: IJID Reg. 2025 Jan 25;14:100577. doi: 10.1016/j.ijregi.2025.100577 (PMC11869006; doi:10.1016/j.ijregi.2025.100577)
Supplement: Supplementary file 1 [file mmc1.docx]

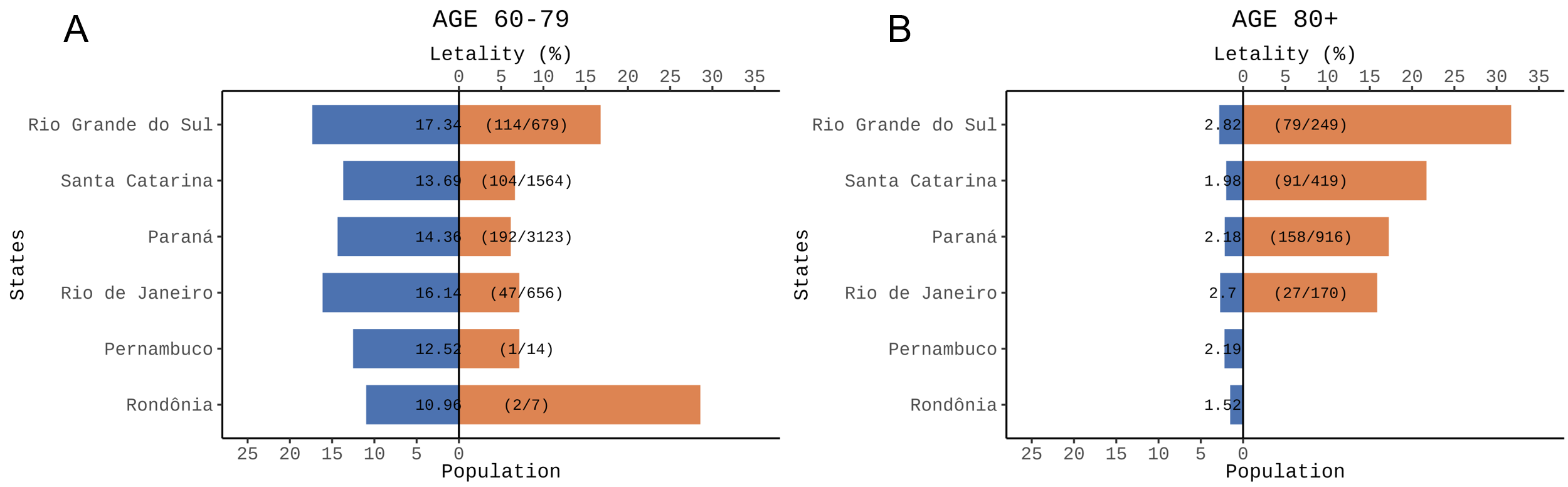


**Supplementary Figure 1** - Elderly population above 60 years old and lethality in cases with warning signs of severe dengue comparison between states. **A** - Focus on the population aged 60–79 years in Rio Grande do Sul (RS), Santa Catarina (SC), Paraná (PR), Rio de Janeiro (RJ), Pernambuco (PE) and Rondônia (RO). The blue bars represent the proportion of the population aged 60–79 years, while the orange horizontal bars represent the proportion of deaths in severe dengue cases (lethality) within this age group in each state. The orange bars also display the number of deaths in severe dengue cases/number of severe dengue cases. **B** - Focus on the population aged 80 years and older in Rio Grande do Sul (RS), Santa Catarina (SC), Paraná (PR), Rio de Janeiro (RJ), Pernambuco (PE) and Rondônia (RO). The blue bars represent the proportion of the population aged 80 years and older, while the orange horizontal bars represent the proportion of deaths in severe dengue cases (lethality) within this age group in each state. The orange bars also display the number of deaths in severe dengue cases/number of severe dengue cases.


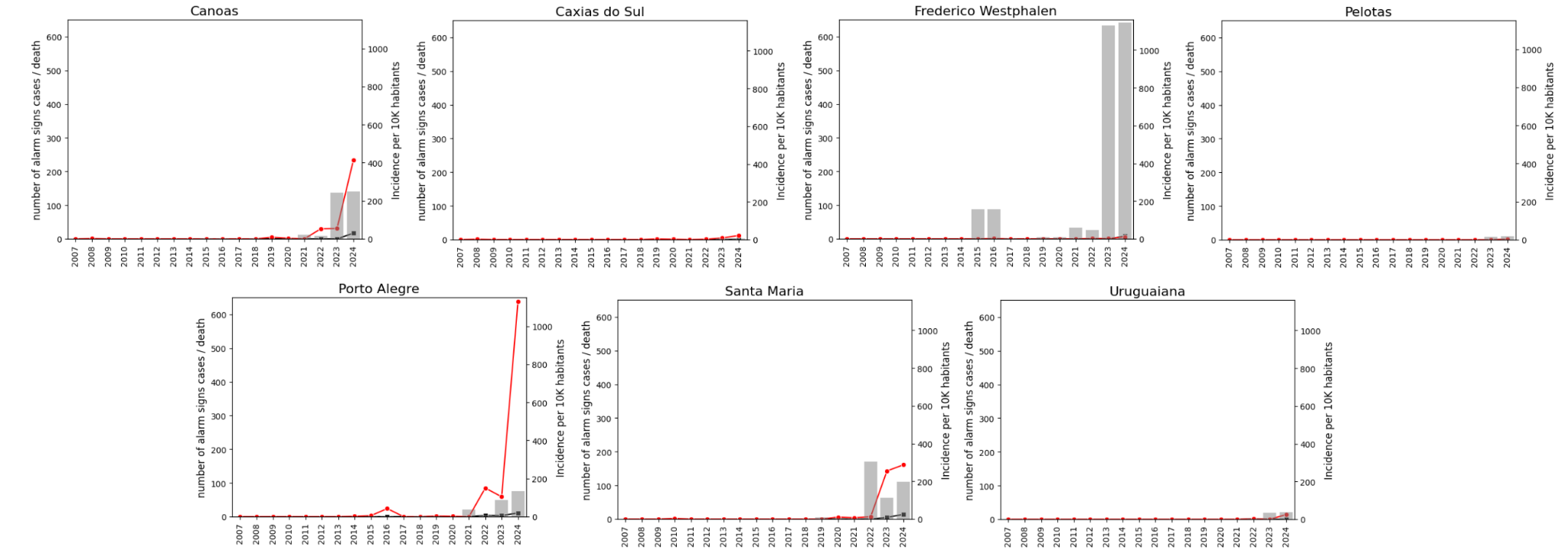


**Supplementary Figure 2** - Incidence per 100K habitants, severe dengue and lethality in severe dengue cases in Canoas (RS), Caxias do Sul (RS), Frederico Westphalen (RS), Pelotas (RS), Porto Alegre (RS), Santa Maria (RS) and Uruguaiana (RS). The bars represent the incidence and the line represents the total numbers of severe dengue (red) and deaths in severe dengue cases (black) per year.


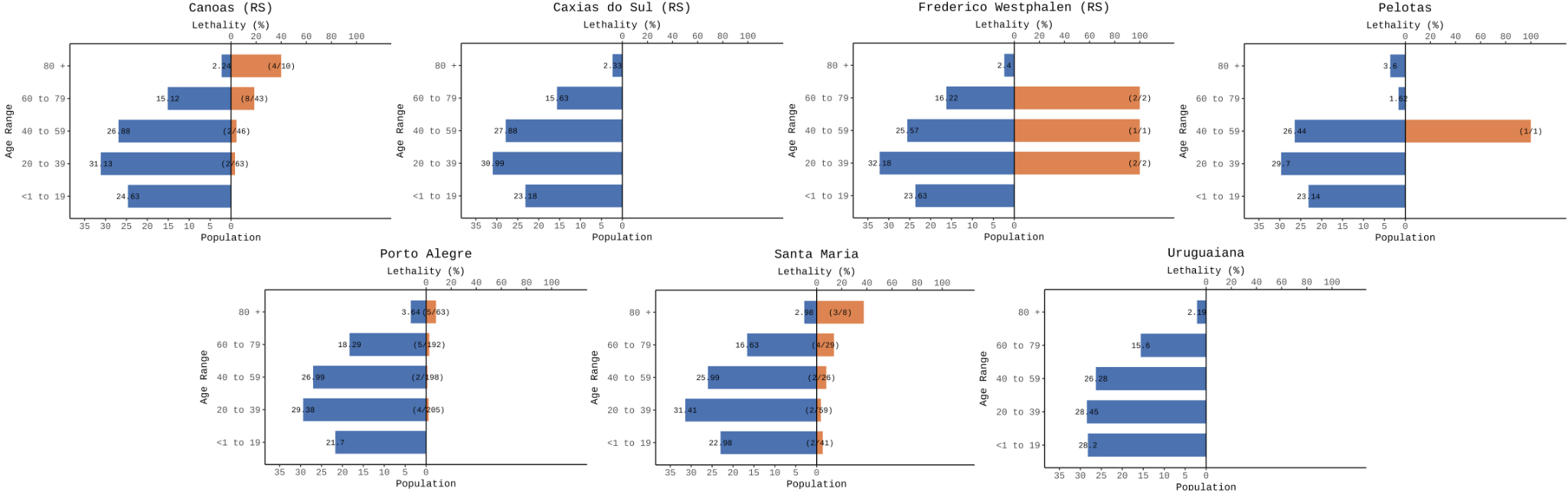


**Supplementary Figure 3** - The pyramid age of Canoas (RS), Caxias do Sul (RS), Frederico Westphalen (RS), Pelotas (RS), Porto Alegre (RS), Santa Maria (RS), Uruguaiana (RS). The blue bars represent the proportion of the population per age group while orange horizontal bars represent the proportion of deaths in severe dengue cases (lethality) per age in each city, over the orange bars there is also the number of deaths in severe dengue cases / number of severe dengue cases.
